# The last 50 years of climate‐induced melting of the Maliy Aktru glacier (Altai Mountains, Russia) revealed in a primary ecological succession

**DOI:** 10.1002/ece3.4258

**Published:** 2018-07-02

**Authors:** Roberto Cazzolla Gatti, Anastasia Dudko, Artem Lim, Alena I. Velichevskaya, Inna V. Lushchaeva, Alice V. Pivovarova, Stefano Ventura, Erica Lumini, Andrea Berruti, Igor V. Volkov

**Affiliations:** ^1^ Bio‐Clim‐Land Centre Biological Institute Tomsk State University Tomsk Russia; ^2^ Department of Geoecology and Geochemistry Institute of Natural Resources Tomsk Polytechnic University Tomsk Russia; ^3^ Institute of Ecosystem Study National Research Council Sesto Fiorentino Italy; ^4^ Institute for Sustainable Plant Protection SS Torino—National Research Council Torino Italy; ^5^ Tomsk State Pedagogical University Tomsk Russia

**Keywords:** Altai Mountains, climate change, deglaciation, glacier, primary succession

## Abstract

In this article, we report and discuss the results obtained from a survey of plants, microorganisms (bacteria and fungi), and soil elements along a chronosequence in the first 600 m of the Maliy Aktru glacier's forefront (Altai Mountains, Russia). Many glaciers of the world show effects of climate change. Nonetheless, except for some local reports, the ecological effects of deglaciation have been poorly studied and have not been quantitatively assessed in the Altai Mountains. Here, we studied the ecological changes of plants, fungi, bacteria, and soil elements that take the form of a primary ecological succession and that took place over the deglaciated soil of the Maliy Aktru glacier during the last 50 year. According to our measurements, the glacier lost about 12 m per year during the last 50 years. Plant succession shows clear signs of changes along the incremental distance from the glacier forefront. The analysis of the plant α‐ and β‐diversity confirmed an expected increase of them with increasing distance from the glacier forefront. Moreover, the analysis of β‐diversity confirmed the hypothesis of the presence of three main stages of the plant succession: (a) initial (pioneer species) from 30 to 100 m; (b) intermediate (*r*‐selected species) from 110 to 120–150 m; and (c) final (K‐selected species) from 150 to 550. Our study also shows that saprotrophic communities of fungi are widely distributed in the glacier retreating area with higher relative abundances of saprotroph ascomycetes at early successional stages. The evolution of a primary succession is also evident for bacteria, soil elements, and CO_2_ emission and respiration. The development of biological communities and the variation in geochemical parameters represent an irrefutable proof that climate change is altering soils that have been long covered by ice.

## 1 INTRODUCTION

The loss of glacier volume has been more or less continuous since the 19th century and was mainly caused by the rise in air temperature (Dyurgerov & Meier, 2000). In many glaciers of the world, the effects of climate change are evident. For instance, in Europe, the warming experienced since the early 1980s in the Alps caused pronounced effects in the glacial and periglacial belts (Haeberli & Beniston, 1998). In Norway, the glaciers investigated by Andreassen, Paul, Kääb, and Hausberg (2008) shrank since the 1930s with an overall area reduction of about 23% for 38 glaciers and of 12% for 164 glaciers since the 1960s. Similarly, in Asia, most Himalayan glaciers have been retreating at a rate that ranges from a few meters to several tens of meters per year (Bajracharya, Mool, & Shrestha, 2007). In Africa, Kilimanjaro and its vanishing glaciers have become an icon of global warming, attracting broad interest (Kaser, Hardy, Mölg, Bradley, & Hyera, 2004). In South America, the tropical Andes show that the glacier retreat, in terms of changes in surface area and length, over the last three decades is unprecedented since the maximum extension of the Little Ice Age (Rabatel et al., 2013). In North America, airborne laser altimetry has been used to estimate volume changes of 67 glaciers in Alaska from the mid‐1950s to the mid‐1990s and this evidenced that the average rate of thickness change was –0.52 m/year (Arendt, Echelmeyer, Harrison, Lingle, & Valentine, 2002).

A recent study (Narozhniy & Zemtsov, 2011) estimated that between 1952 and 2008, the glacier area in different regions of the Altai Mountains, in Western Siberia, has decreased by 9%–27% and the volume by 12%–24%. From 1952 to 2006, the total glacier area in the Aktru basin (Altai Mountains) shrank by 7.2%, corresponding to 1.2 km^2^ (Surazakov, Aizen, Aizen, & Nikitin, 2007). In particular, the valley glacier Maliy Aktru (“Small Aktru”) showed a loss of 8.6% (1952–2006) and its retreatment is one of the best documented because of the constant positioning of benchmarks on the ground by local researchers. The Maliy Aktru deglaciation seems to be strongly related with the fact that the mean annual temperature in the Altai Mountains has increased of 1.3–1.7°C in the last 50 years (Mandych et al., 2012).

In fact, together with variations in tree growth (Battipaglia et al., 2015), changes in water salinity (Banerjee, Cazzolla Gatti, & Mitra, 2017), and raising of water sea level (Galbraith et al., 2002), deglaciation is a key indicator of global climate change (Haeberli, Hoelzle, Paul, & Zemp, 2007). Satellite analyses have been mainly used to assess the loss of surface and volume of glaciers worldwide (Paul, Kääb, Maisch, Kellenberger, & Haeberli, 2004). However, to better understand the biological effects of climate change, the ground analyses offer the possibility to study the consequences of deglaciation as revealed by primary ecological successions (Chapin, Walker, Fastie, & Sharman, 1994). The exposure of deglaciated soils provides opportunities for the development of microbial, fungal, and plant communities. These communities exert several important ecological functions and constitute a significant part of vegetation at glacier forefronts and high latitudes. In fact, biocrusts are often distributed in extreme habitats, such as the Polar Regions, and have important influences on soil and higher plants (Hodkinson, Webb, & Coulson, 2002). For instance, biological soil crusts formed by phototrophic organisms were investigated on Arctic Svalbard in Norway (Borchhardt, Baum, Mikhailyuk, & Karsten, 2017) and on Livingston Island (Williams et al., 2017).

Recently, deglaciated soils are colonized by a diverse community of bacteria and fungi even during the first 4–5 years following glacial retreat (Schmidt et al., 2008). Photosynthetic and nitrogen‐fixing bacteria play important roles in acquiring nutrients and facilitating ecological succession in soils near some of the highest elevation receding glaciers. Nemergut et al. (2007) studied the microbial community succession over deglaciated soils and showed that evenness, phylogenetic diversity, and the number of phylotypes were lowest in the youngest soils, increased in the intermediate‐aged soils, and plateaued in the oldest soils. Different other studies (Gobbi, Bernardi, Pelfini, Rossaro, & Brandmayr, 2006; Fernández‐Martínez et al., 2017; Jones & Henry, 2003; Raffl, Mallaun, Mayer, & Erschbamer, 2006; Tscherko et al. 2008) reported the evidence of primary successions of plants, bacteria, nutrients, etc., along glacier foreland chronosequences. Two main issues emerged from these research studies: First, that primary succession in these high Arctic sites is strongly controlled by local environmental conditions, and second, that most of the analyzed communities can be grouped according to early and late‐colonizing species.

Even if there is some evidence of an ecological succession over deglaciated soils in different part of the world, except for some local reports (see, for instance, Volkova & Volkov, 2014, in Russian) the ecological effects of deglaciation have been poorly studied and have not been quantitatively assessed at the Maliy Aktru glacier in the Altai Mountains. Here, we studied the ecological changes of plants, fungi, bacteria, and soil elements that take the form of a primary ecological succession and that took place over the deglaciated soil of the Maliy Aktru glacier during the last 50 year. Our hypothesis is that glacier melting offers the opportunity to the development of an ecological primary succession and we checked whether and what seral stages of this succession develop as far as we move from the glacier forefront (i.e., during the last 50 years of deglaciation). Moreover, we explored the possibility to identify defined biological communities along the deglaciation profile.

## 2 MATERIALS AND METHODS

### 2.1 Study area

**Figure 1:**
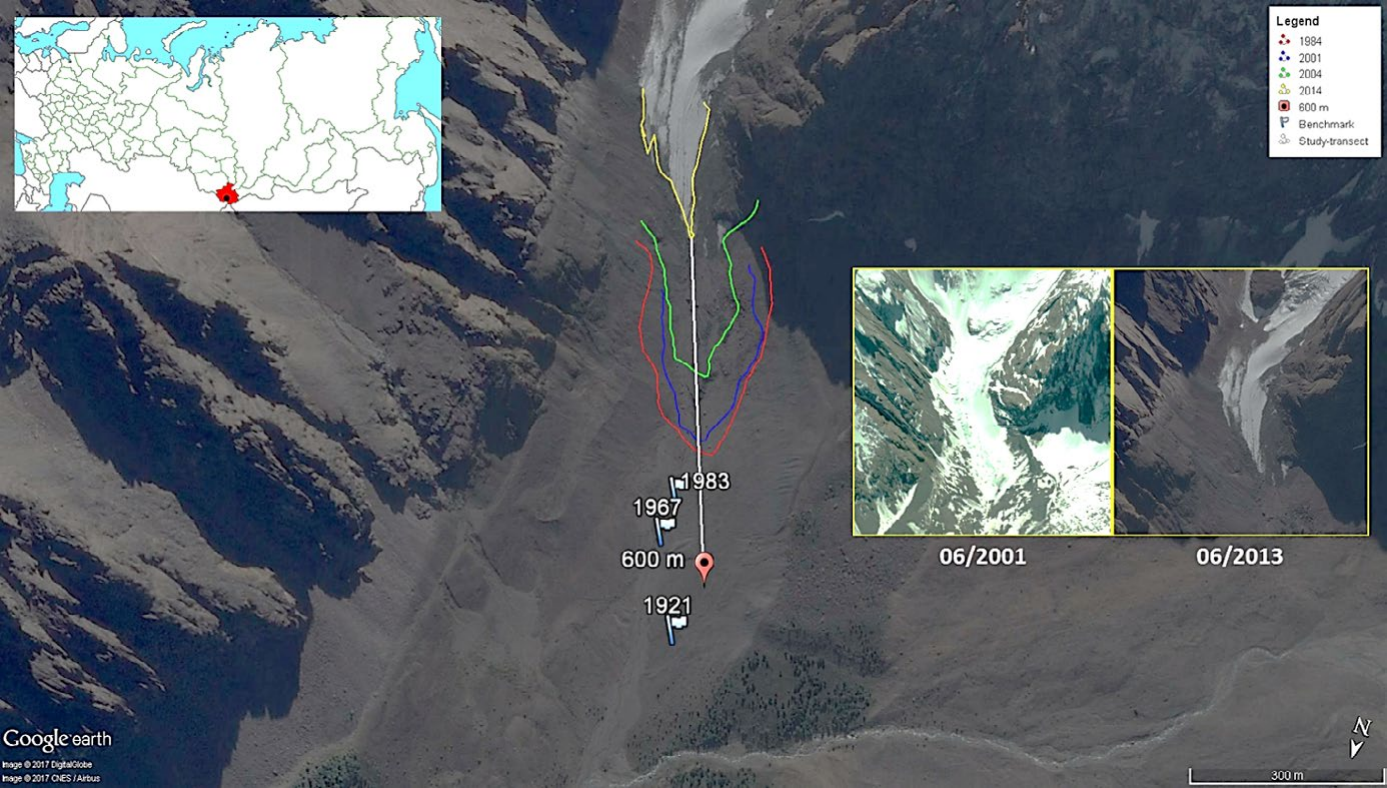
Study area. Flags represent the benchmarks left overs rocks by different researchers during the last century. Colored lines define the glacier's retreatment as shown by satellite images. Red point is the sampling limit of this study (600 m white transect from the current glacier forefront). Changes in ice‐covered surface from June 2001 to June 2013 are shown in the two smaller images (Landsat Imagery‐Google Earth)

The glacier Maliy Aktru (Figure 1) belongs to the mountain–glacial pool of Aktru and is located on the Northern slope of the Severo‐Chuyskiy range (Central Altai). The basin represents a closed watershed of the upper courses of glacial river Aktru (Mandych et al., 2012). The climate of the basin is characterized by low temperatures (annual average −5.2±°C, summer average +8.7±°C) and high diurnal temperature variation (15–20°C). The pool is considered to be climatically representative of the Altai and includes seven glaciers (Mandych et al., 2012).

The Maliy Aktru glacier is oriented in the north‐western direction and descends from 3,710 to 2,240 m. Due to its large opacity, the glacier is very stable. The position the glacier's snout, in the period of its maximum advance in the middle of the nineteenth century, is well recorded with high terminal moraine formation (that time the glacier ended at an altitude of 2,200 m and had a length of 4.8 km). In the last 150 years, the retreat and the reduction in its size were uneven but relevant (Narozhniy & Zemtsov, 2011).

The young moraines of the glacier are located at a height of 2,200–2,250 m above sea level (Narozhniy & Zemtsov, 2011), have a northern exposure, falls away to the upper part of the mountain‐forest zone, and are surrounded by diverse plant communities (forests, petrophyte community on rocks, talus, stone placers, etc.). The topography is represented by moraines, meso‐depressions, outwash sites, and slopes. The granulometric composition of the sediments varies from large to fine soil fraction. The moraines are composed of boulders and gravel, with a small quantity of fine‐grained deposits (Narozhniy & Zemtsov, 2011). Soils on the young moraines of the glacier are composed of cryosols and podzols. In the moraine, the organogenic horizon (2–3 cm) is formed over 60‐year‐old fragments (Narozhniy & Zemtsov, 2011).

### 2.2 Plant sampling

Starting from the glacier forefront, we set a linear transect of 600 m toward the north. Along the transect, in the first 120 m from the glacier forefront (corresponding to the 2006 glacier extension), we made an extensive survey of all herbs, mosses, and trees (of any size) falling within 5 m on each side of the transect (covering a width of 10 m). Thus, the total surveyed surface was of 10 × 120 m, that is, 1,200 m^2^. We listed all the species along with their abundances.

Then, since 120 m from the glacier forefront, we sampled at 150 m and every 50 m along the transect (up to 600 m—corresponding to the 1966 glacier extension—for a total of 10 sampling points along the transect). We marked GPS coordinates and altitude and measured the linear distance with a measurement tape. At each sampling point, with a square frame of 2 × 2 m, we sampled in a first plot cantered over the transect and in other two on each side of the transect (for a total of five plots, with a total sampled area of 10 m^2^ per sampling point). As above, at these sampling points, we collected data on species and their abundances.

### 2.3 Soil respiration, sampling, and granulometry

From the glacier forefront (0 m) to 600 m, we collected two spatially separated soil samples every 50 m (for a total of 13 × 2 samples) with a small shovel. We put the collected soil in plastic buckets. We mixed the soil thoroughly in the bucket, breaking up all cores. Then, we filled the soil bags discarding any extra soil and stones. We preserved the soil samples in a thermic bag before analyzing their granulometry and microelements concentration (see below) and stored them at −20°C for DNA extraction.

At each distance (50 m) from the glacier forefront where we collected soil samples and in five points horizontally differentiated (at a minimum distance of 2 m each), we measured soil CO_2_ emissions (in ppm) and respiration (in gCO_2_ m^−2^ hr^−1^) with a PP System EGM analyzer. We then calculated a mean and a *SE* CO_2_ emission and respiration every 50 m from 0 to 600 m.

For granulometric analyses, we reduce all representative air‐dried soil samples to a size of 100 g. Then, we sift the soil through a sieve set (five sieves: size 2 mm; 0.5 mm; 0.25 mm; 0.1 mm; 0.04 mm). After the shaking was completed, the material on each sieve was weighed. The weight of the sample of each sieve was then divided by the total weight to give a percentage retained on each sieve following the formula:

$$\%\text{Retained}=\frac{W_{\mathrm{Sieve}}}{W_{\mathrm{total}}}\times 100,$$

where *W*_Sieve_ is the aggregate weight in the sieve and *W*_Total_ is the total weight of the aggregate (Bedaiwy, 2012; Laker & Dupreez, 1982).

### 2.4 Ca, Hg, C, and N soil concentration

Calcium (Ca) and Mercury (Hg) analyses were made in the laboratory of Geoecology and Geochemistry Department of Tomsk Polytechnic University (Tomsk, Russia). Ca concentration was measured by Neutron Activation Analysis (NAA), a nuclear process used for determining the concentrations of chemical elements. The sample was bombarded with neutrons, causing the elements to form radioactive isotopes. Because the radioactive emissions and radioactive decay are well known for each element, we studied the emissions spectra of the radioactive sample of calcium (Obrusnik, 1984; Win, 2004). Hg content was determined by atomic absorption spectroscopy (AAS; Welz & Sperling, 2008). The analysis was performed with the mercury analyzer RA 915+ PYRO – 915+. AAS is a spectro‐analytical procedure for the quantitative determination of chemical elements using the absorption of optical radiation (light) by free atoms in the gaseous state (García & Báez, 2012). In the resonance transition frequency, atoms absorb light selectively and pass to an excited state. Nitrogen (N) and carbon (C) concentrations in soil samples were measured by “ThermoFlash 2000 NC soil” after dry combustion at 900°C and Cu catalyzed (Batjes, 1996).

### 2.5 Microorganisms (bacteria and microfungi) sampling, growth and classification

At each sampling point (*n* = 26: one every 50 m from 0 to 600 m) we collected two spatially separated soil samples with a sterilized shovel and we sifted them with a sterilized sieve (1 mm mesh). The resulting soil was preserved in sterilized phials and stored in thermic bags with ice to keep the temperature below 5°C before reaching the laboratory.

Cell suspensions were prepared from 5 g soil dispersed in 45 ml sterile 0.9% NaCl for 15 min on a shaker. We used low‐speed centrifugation (2 min at 2,000 rpm) in order to decrease the number of soil particles in the supernatant. The bacterial suspension was analyzed for (a) viable culturable bacteria enumeration on agar plates; (b) total bacterial number count; and (c) Biolog^®^ tests of metabolic activity.

The eutrophic bacteria were measured via agar plate counting using diluted soil suspensions on a pancreatic digest of fish flour (DFF or MPA) of the following composition: 8 g/L DFF, 8 g/L peptone, 4 g/L NaCl, 20 g/L agar–agar at pH of 7.0 to 7.4. The oligotrophic bacteria were detected via agar plate counting using diluted soil suspensions (20 g/L in distilled water). The Actinobacteria (former actinomycetes) were detected via agar plate counting using diluted soil suspensions on starch–ammonium medium (SAM) of the following composition: 10.0 g/L soluble starch, 2.0 g/L (NH_4_)_2_S0_4_, 1.0 g/L K_2_NP0_4_, 1.0 g/L MgSO_4_‐7H_2_O, 1.0 g/L NaCl, 3.0 g/L CaCO_3_, 20 g/L agar–agar, 1,000 ml tap water. The media were sterilized at 120°C for 20 min. The microfungi were measured via agar plate counting using diluted (1:10) soil suspensions of 100 ml on Czapek–Dox agar. The media was sterilized at 112°C for 30 min. The inoculated agar plates were incubated in a dark environment at 20°C for 7–10 days.

The coefficient of mineralization was calculated as the ratio of the number of microorganisms able to assimilate inorganic sources of nitrogen (Actinobacteria) and to the number of eutrophic bacteria, able to assimilate organic nitrogen. The mineralization coefficient allows evaluating the microbial activity on the organic matter mineralization processes in the soil (Zvyagintsev, 1991).

The coefficient of oligotrophy was calculated as the ratio of the number of oligotrophic microorganisms able to assimilate limited concentrations of nutrients in the soil and the number of eutrophic bacteria that, on the contrary, develop better on relatively rich organic substrates. The coefficient of oligotrophy allows assessing the degree of soil organic enrichment available to eutrophic microorganisms (Mishustin, 1975).

The total number of bacterial cell in the soil samples was measured using microscopic count with LIVE/DEAD BacLight kit (Invitrogen). A Biolog EcoPlate (Biolog Inc., USA) containing 31 different carbon sources was used to assess the average well color development (AWCD). A list of the 31 substrates tested in this work is provided in Supporting Information Table S1. The Biolog plates were inoculated with a 150 μl soil suspension (from a concentration of 25 ml of the bacterial culture by centrifugation at 10,000 *g* for 10–15 min; 1 ml of this suspension was then added to 30 ml centrifuge tubes with 20 ml of 0.85% NaCl for live bacteria or 20 ml of 70% isopropyl alcohol for killed bacteria; the pellets were resuspended and centrifuge again with 10 ml of the previous buffers) and incubated at 20°C in the darkness for 14 days. The color of the cells was measured every day at an optical density of 590 nm, using the Multiscan FC microplate photometer (Thermo Scientific, China). Based on the recorded color development of each triplicate plate, the AWCDs were calculated daily. Kinetic curves were constructed to reflect the AWCD as a function of time. For the comparison of communities from different soils, a standard AWCD value in the middle of the exponential growth phase of the response curve was used following previous recommendations (Preston‐Mafham, Boddy, & Randerson, 2002; Schultz & Ducklow, 2000).

### 2.6 DNA extraction, PCR amplification, and amplicon sequencing

Soil DNA was extracted using the MoBio Power Soil DNA isolation kit (MoBio Laboratories, Carlsbad, CA, USA) following manufacturer's instructions, and concentration of purified DNA was quantified with a Qubit^®^ 2.0 Fluorometer (Invitrogen, Grand Island, USA).

In order to investigate the total fungal community, the nuclear ribosomal ITS2 region was amplified using Hot Star Taq DNA polymerase (Qiagen) from all DNA extracts by means of a seminested PCR approach. In the first PCR, the entire ITS (ITS1‐5.8S‐ITS2) region was amplified with the generic fungal primer pair ITS1F‐ITS4 (Gardes & Bruns, 1993; White, Bruns, Lee, & Taylor, 1990). The cycling conditions were: an initial step at 95°C for 15 min, 35 cycles at 95°C for 35 s, 57°C for 35 s, 72°C for 45 s, and a final extension step of 72°C for 7 min. Each PCR product was checked on an agarose gel, diluted at 1:20 and used as template (*n* = 56) in the seminested PCR with primer fITS9‐ITS4 (Ihrmark et al., 2012), targeting the ITS2 region, and added to Illumina overhang adapter sequences. Forward overhang: 5′‐TCGTCGGCAGCGTCAGATGTGTATAAGAGACAG‐[locus specific target primer], Reverse overhang: 5′ GTCTCGTGGGCTCGGAGATGTGTATAAGAGACAG‐[locus specific target primer]. The seminested PCR cycling conditions were: an initial step at 95°C for 15 min, 27 cycles at 95°C for 30 s, 57°C for 30 s, 72°C for 30 s, and a final extension step of 72°C for 7 min. All PCRs were performed using a T3000 thermal cycler (Biometra GmbH, Gottingen, Germany). PCR products were checked on an agarose gel, and the three replicates of each sample were pooled and purified using the Wizard SV Gel and PCR Clean‐Up System (Promega) following the manufacturer's instructions and then sequenced using the Illumina MiSeq technology. The paired‐end (2 × 300 bp) sequencing was performed by BMR Genomics, Padua, Italy.

### 2.7 Bioinformatics

Raw sequences were treated with the freeware mothur v1.33 (Schloss et al., 2009). After making contigs, ITS2 rRNA raw sequences were filtered based on the following specifications: minimum fragment length of 200 bp, the absence of ambiguous nucleotides, and maximum 10‐bp‐long homopolymers. Sequences were then clustered based on 100% similarity and singletons were removed from the dataset. Potential chimeric sequences were identified de novo and removed using the open‐source UCHIME algorithm (Edgar, Haas, Clemente, Quince, & Knight, 2011). Sequences were then clustered de novo into Operational Taxonomic Units (OTUs) at 97% similarity using the OptiClust method (Westcott & Schloss, 2017). Pruning of OTUs with low numbers of sequences (<10) was carried out on a per‐sample basis and the most abundant sequence of each OTU was selected as the representative. Taxonomy was assigned through a search for similar sequences conducted with Blast v2.2.29 (Zhang, Schwartz, Wagner, & Miller, 2000) against the v2.7 release of the UNITE + INSD online database (Abarenkov et al., 2010). Plants, Chromista, Protista, Metazoa, Protozoa, and unidentified sequences (found in only one sample and with <80% max ID and/or <75% coverage with UNITE + INSD database fungal accessions) were removed from the dataset. At this point, rarefaction curves were computed in order to evaluate the sequencing efforts provided. As a normalization step to reduce bias associated with different sequencing depths, all samples were subsampled down to ~80% of the size of the smallest sample. Trophic modes and ecological guilds of each OTU were annotated using the online tool FUNGuilds (Nguyen et al., 2016).

### 2.8 Statistical analyses

We checked for possible correlations between the distance from the glacier forefront and plant richness and abundance, soil characteristics, concentration of minerals and microorganisms by calculating Spearman's correlation coefficient (Spearman, 1904). Then we run a linear regression analysis, setting significant values at *p* < 0.05 and highly significant ones at *p* < 0.01. Residuals were evaluated for the normal distribution of their variance, their drift from the order of data and their independence from the time (since our data derived from a time series). A Cook's Distance statistics (Cook, 1977) was performed to find influential outliers with a threshold of *D* < 4/[*n* = 22]. Plant α and β‐diversity were analyzed with EstimateS software (Colwell, 1997).

All statistical analyses concerning fungal community diversity and structure were done using R v3.2.0 (http://cran.r-project.org/). The R packages vegan (Oksanen et al., 2015) was mainly adopted.

A 2D Nonmetric Multidimensional Scaling (NMDS) biplot based on the Bray–Curtis dissimilarity matrix was constructed to graphically assess the differences in the community composition along the gradient of distance from the glacier forefront. Measured environmental variables including spatial variables, soil variables, and plant types were plotted as vectors in the two NMDS and their squared correlation coefficient was calculated as an indicator of goodness of fit, to assess their potential role in the community structuring and composition. In order to quantify the fractions of fungal community variance explained by all measured environmental variables, the partition of variation was performed. Significant variables were forward‐selected from three subsets (spatial variables, soil variables, and plant types) in order to avoid explanatory variable collinearity in the model and to search for parsimony, following the methods in Borcard, Gillet, and Legendre (2011). Subsequently, the fractions of fungal community variance independently explained by the forward‐selected environmental variables were computed and tested for significance. Finally, a correlation network based on Spearman's coefficient was computed using fungal trophic modes, plant types and diversity indices, soil variables, and the distance from the glacier forefront as input.

## 3 RESULTS

### 3.1 Plants succession and diversity

In the first 120 m from the glacier forefront, on a survey area of 1,200 m^2^ (10 × 120 m) we collected 928 individuals of herbs, mosses, and trees belonging to 33 species. In the other plots (10 m^2^ each every 50 m, for a total sampled area of 100 m^2^), from 150 to 600 m, we collected a total of 1,194 individuals belonging to 60 species, whose 18 trees, six mosses, and 61 herbs. In total, we collected 2,122 individuals of 86 species.

**Figure 2:**
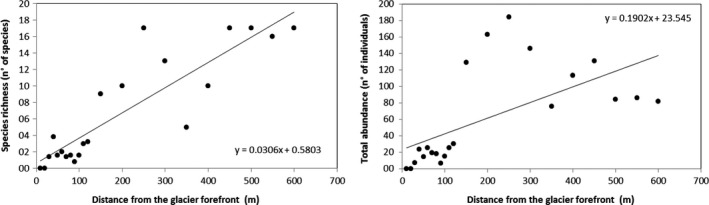
Species richness (species/10 m^2^, left panel) and total abundance of species (number/10 m^2^, right panel) plotted against the distance from the glacier forefront. Linear regression line's equation is shown

The correlation (Figure 2) between the distance from the glacier forefront was high with both species richness and abundance (Spearman's *r* = 0.88, *p* < 0.01; *r* = 0.79 *p* < 0.01, respectively).

Regression analysis between distance and (a) species richness (*F* = 70.63, *p* < 0.01); and (b) species abundances (*F* = 11.77, *p* < 0.01) was highly significant. Residuals analysis showed normal distribution, constant variance and independence over time (with no influential outlier; Cook's *D* < 4/[*n* = 22]).

**Figure 3:**
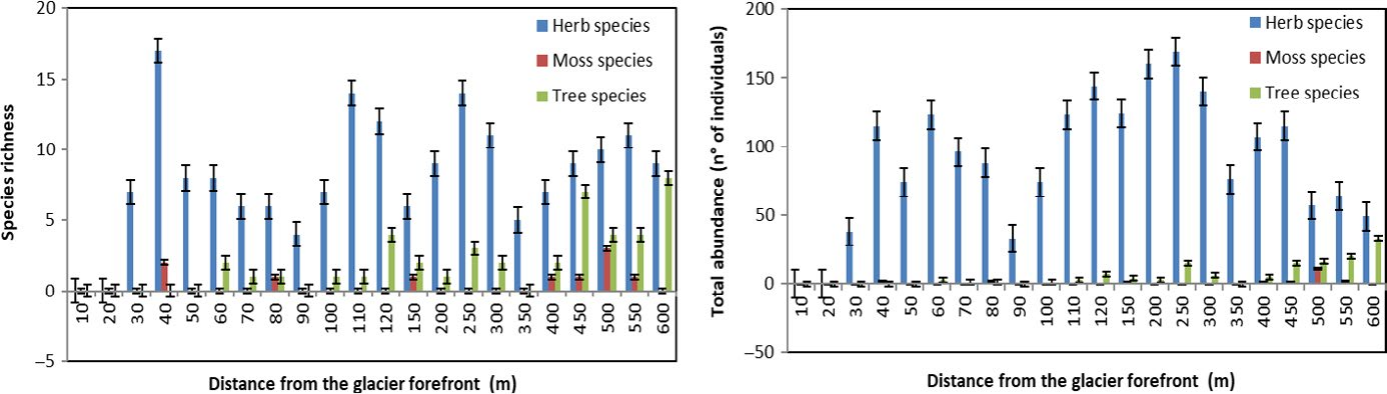
Histograms of species richness (left) and abundance (right) along incremental distance from the glacier forefront. Mean values ± *SE*

**Figure 4:**
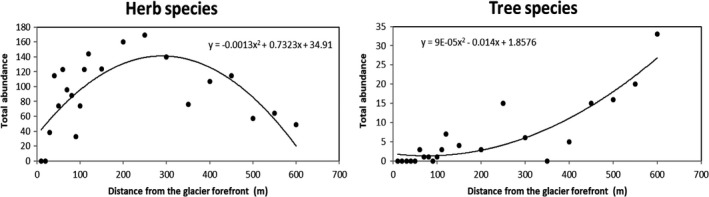
Abundances of herb (left) and tree (right) species along an incremental distance from the glacier forefront. The equations of the hump‐shaped (grass species) and exponential (tree species) best fitted curves are shown

The first 20 m from the glacier forefront were devoid of any plant species. After 20 m, grass species richness remained almost stable along incremental distance, while tree species richness increased (Figure 3). Instead, grass species abundance showed an hump‐shaped trend (*R* ^2^ = 0.51) with a decrease when the tree species abundance, which showed an exponential trend (*R* ^2^ = 0.79), increased (Figures 3 and 4).

**Figure 5:**
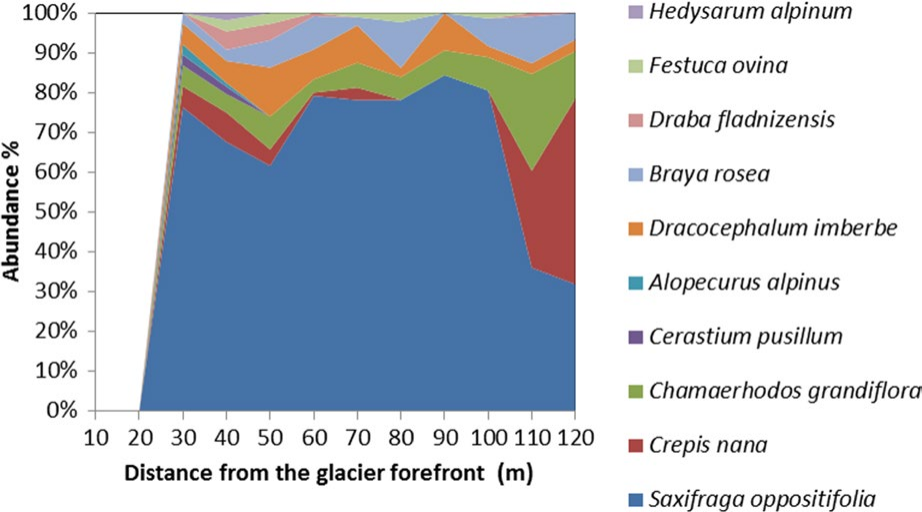
Abundance (%) of the 10 most dominant (*n*_0–120 m_ > 2) species in the first 120 m from the glacier forefront

In the first 120 from the glacier forefront, the most abundant species was *Saxifraga oppositifolia* and only 10 species accounted for 95% of the total abundance (Figure 5). No tree species were detected in the first 50 m and only 16 juvenile trees (height <5 cm) belonging to *Juniperus pseudosabina, Salix nummularia, Salix saposchnikovii, Myricaria dahurica, Salix hastata* and *Salix reticulate*, were detected in the first 120 m. 12 other tree species started to show up since 150 m from the glacier forefront. The maximum amount of trees was in the last 200 m of our transect (400–600 m) where *Myricaria dahurica* and *Salix hastata* were dominant (17 and 68 individuals, respectively).

The analysis of the development of Shannon and Simpson's *α*‐diversity indices along the incremental distance from the glacier forefront (Table 1) shows that both increase form values of 1.50 and 3.28 to 2.20 and 4.54 at 600 m, respectively. Chao 1 index, which is considered a measure of the effective number of species, also shows (Tab. 1) an increment from 20.23 to 144.47 species.

**Table 1:** The development of Chao 1, Shannon, and Simpson (1/*D*) indices of α‐diversity along incremental distance from the glacier forefront

α‐diversity						
Distance (m)	Chao 1 mean (S cumulated)	Chao 1 (S cumulated) *SD*	Shannon mean	Shannon *SD*	Simpson inv mean	Simpson inv *SD*
10	20.23	0.00	1.50	0.50	3.28	1.64
20	40.34	22.50	1.65	0.41	3.61	1.55
30	58.78	33.53	1.80	0.34	4.01	1.38
40	67.29	32.50	1.88	0.28	4.16	1.07
50	79.94	37.05	1.94	0.22	4.30	0.87
60	83.81	32.69	1.97	0.20	4.33	0.74
70	90.02	32.50	1.99	0.19	4.35	0.67
80	98.35	33.42	2.00	0.18	4.39	0.64
90	100.87	30.90	2.02	0.16	4.38	0.53
100	104.11	28.19	2.03	0.15	4.40	0.49
110	107.97	27.89	2.05	0.14	4.44	0.45
120	112.48	27.51	2.05	0.12	4.44	0.38
150	116.99	27.43	2.06	0.11	4.48	0.36
200	120.82	27.64	2.06	0.10	4.47	0.31
250	125.36	27.83	2.07	0.09	4.48	0.28
300	129.02	27.46	2.08	0.08	4.50	0.24
350	133.67	28.20	2.08	0.07	4.51	0.21
400	135.95	27.76	2.09	0.06	4.53	0.18
450	139.08	27.93	2.09	0.05	4.53	0.15
500	142.24	27.77	2.10	0.04	4.54	0.11
550	143.71	27.02	2.10	0.03	4.53	0.08
600	144.47	25.93	2.10	0.00	4.54	0.00

Beta‐diversity was analyzed for the evaluation of shared species and species complementarity (by the Morisita–Horn index) between pairs of samples at each point along the transect from 0 to 600 m. Shared species (Table 2) and Morisita–Horn index (Table 3) values are shown in a matrix of comparison between sampling distances.

**Table 2:**
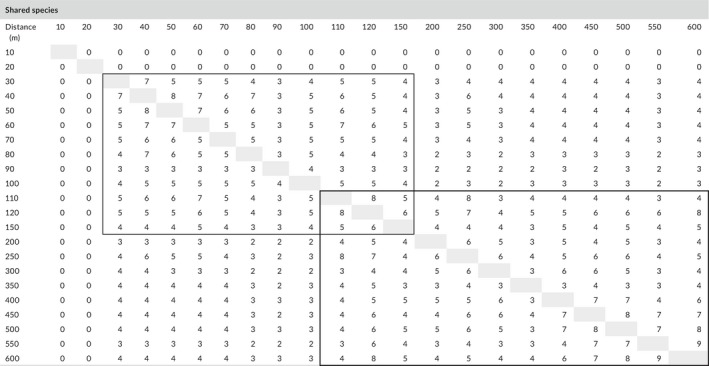
Matrix of shared species (β‐diversity) between pairs of sampling points along incremental distance from the glacier forefront. Two main blocks of shared species (threshold >2) are underlined

**Table 3:**
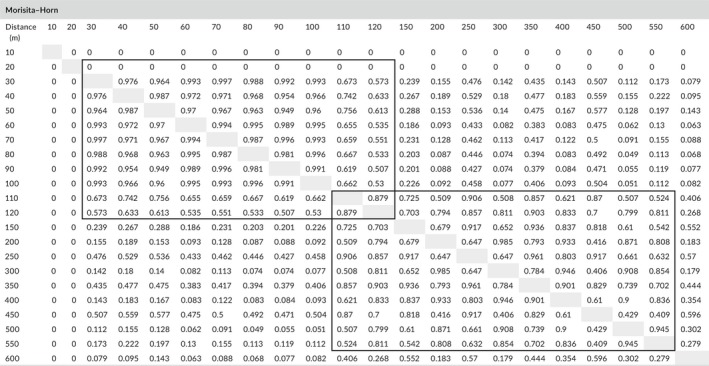
Matrix of the Morisita–Horn species complementarity index (β‐diversity) between pairs of sampling points along incremental distance from the glacier forefront. Two main blocks of complementarity (threshold >0.4) are underlined

### 3.2 Soil properties succession

Soil granulometry did not show any specific trend (Supporting Information Figure S1), although the fraction of smaller particles (<0.5 mm) increase from 150 to 300 m from the glacier forefront where the slope slightly decreases toward small valleys.

**Figure 6:**
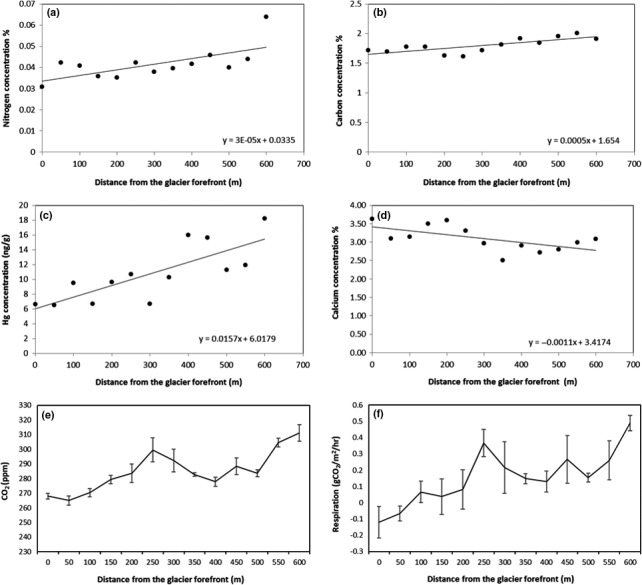
(a) Concentration of nitrogen (%), (b) carbon (%), (c) mercury (ng/g), (d) calcium (%), (e) soil CO_2_ emission (ppm), and (f) respiration (gCO_2_ m^−2^ hr^−1^) along incremental distance from the glacier forefront

Soil nitrogen and carbon were positively correlated with the distance from the glacier forefront (Spearman's *r* = 0.59, *p* = 0.03; *r* = 0.74, *p* < 0.01, respectively). Linear regression analysis (Figure 6) shows a significant trend (*F* = 8.81, *p* = 0.01) for nitrogen and a highly significant trend (*F* = 14.71, *p* < 0.01) for carbon. Both increased along the incremental distance from the glacier forefront (Figure 6).

A similar high significant incremental trend (*r* = 0.89; *F* = 18.20, *p* < 0.01) is shown for mercury concentration (Figure 6). Calcium concentration, instead, shows a significant decreasing trend (Figure 6) along the incremental distance from the glacier forefront (*r* = −0.67; *F* = 6.52, *p* = 0.03). Residuals analysis for these four elements showed normal distribution, constant variance, and independence over time (with no influential outlier, Cook's *D* < 4/[*n* = 13]). Concentration values at each sampling distance are reported in Table 4.

**Table 4:** Carbon, nitrogen, calcium (all in %), and mercury (ng/g) concentration (mean value and standard error, *SE*) along incremental distance from the glacier forefront

Distance	*N* (%)	*SE*	C (%)	*SE*	Hg (ng/g)	*SE*	Ca (%)	*SE*
0	0.031	0.002	1.72	0.09	6.60	0.36	3.64	0.20
50	0.042	0.002	1.70	0.09	6.50	0.36	3.10	0.17
100	0.041	0.002	1.78	0.10	9.50	0.52	3.14	0.17
150	0.036	0.002	1.78	0.10	6.65	0.37	3.50	0.19
200	0.035	0.002	1.63	0.09	9.60	0.53	3.60	0.20
250	0.042	0.002	1.62	0.09	10.70	0.59	3.32	0.18
300	0.038	0.002	1.72	0.09	6.70	0.37	2.97	0.16
350	0.040	0.002	1.82	0.10	10.25	0.56	2.51	0.14
400	0.042	0.002	1.92	0.11	15.97	0.88	2.91	0.16
450	0.046	0.003	1.84	0.10	15.65	0.86	2.72	0.15
500	0.040	0.002	1.96	0.11	11.25	0.62	2.80	0.15
550	0.044	0.002	2.01	0.11	11.90	0.65	2.99	0.16
600	0.064	0.004	1.91	0.11	18.20	1.00	3.09	0.17

Similarly, soil CO_2_ emission and respiration show a positive increment along the incremental distance from the glacier forefront (Figure 6).

### 3.3 Bacteria and fungi (Abundance, Type, AWCD)

**Figure 7:**
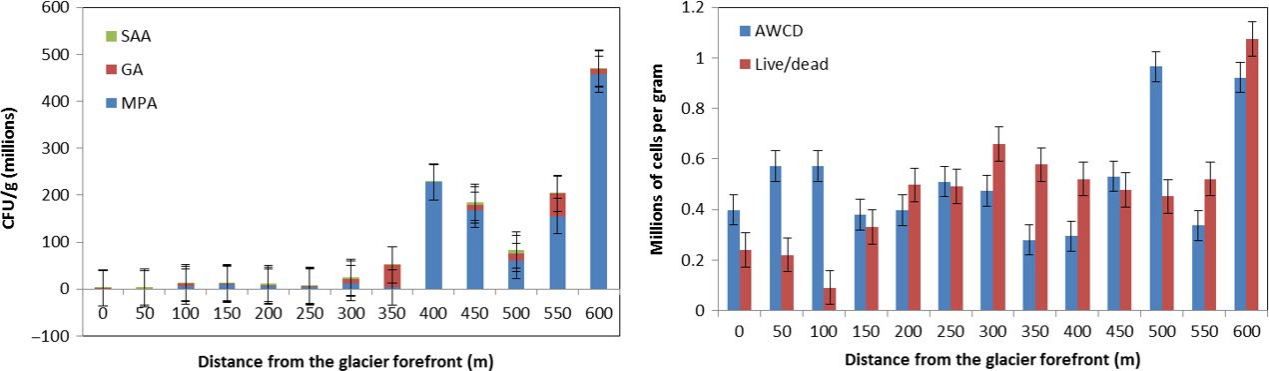
Microbiological trends along incremental distance from the glacier forefront (CFU = colony‐forming unit; MPA = meat–peptone agar; SAA = starch–ammonium agar; GA = growth on agar–agar; AWCD = average well color development). Mean values ± *SE*

The number of Colony‐Forming Unit (CFU) of microorganism increased significantly (*F* = 17.60, *p* < 0.01) along the incremental distance from the glacier forefront, as shown in Figure 7 by their growth over three different terrains (MPA = meat–peptone agar; SAA = starch–ammonium agar; GA = agar–agar). A similar positive significant trend (*F* = 11.61, *p* < 0.01) was observed in the live/dead viability assay, but not (*F* = 1.57, *p* = 0.24) in the average well color development (AWCD; Figure 7). Residuals analysis showed normal distribution, constant variance and independence over time in the case of CFU and live/dead viability assay (with only the last points at 600 m as influential outliers; Cook's *D*_*n* = 13, 600 m_ = 1.35 and 0.98, *p* < 0.05, respectively). Two influential outliers were detected in the final points of the succession (500 and 600 m) of the AWCD trend (*D*_*n* = 13, 500–600 m_ > 0.31, *p* < 0.05).

Bacterial and fungal growth (Table 5) showed a linear regression trend along incremental distance from the glacier forefront (m), which is highly significant for eutrophic (*F* = 10.19, *p* < 0.01) and culturable (*F* = 12.32, *p* < 0.01) bacteria, significant for microfungi (*F* = 5.94, *p* = 0.03) and nonsignificant for oligotrophic bacteria (*F* = 4.84, *p* = 0.05) and Actinobacteria (*F* = 0.11, *p* = 0.74). In the significant regression trends, residuals distribute normally, with a constant variance and a Cook's *D* < 4/[*n* = 13].

**Table 5:** Growth (% cells/g, mean value and standard error, *SE*) of eutrophic and oligotrophic bacteria, actinobacteria, microfungi and culturable bacteria, and oligotrophication (OR) and mineralization (MR) rates along incremental distance from the glacier forefront

Distance (m)	Eutrophic	*SE*	Oligotrophic	*SE*	Actinobacteria	*SE*	Microfungi	*SE*	Culturable	*SE*	OR	*SE*	MR	*SE*
0	13.12	0.72	12.68	0.70	12.51	0.69	0.00	0.00	14.65	0.81	0.59	0.03	0.50	0.03
50	12.61	0.69	13.14	0.72	15.13	0.83	0.00	0.00	15.33	0.84	1.70	0.09	12.47	0.69
100	15.48	0.85	15.51	0.85	14.79	0.81	7.60	0.42	16.41	0.90	1.03	0.06	0.50	0.03
150	16.10	0.89	14.45	0.79	14.54	0.80	6.91	0.38	16.44	0.90	0.19	0.01	0.21	0.01
200	15.48	0.85	14.90	0.82	14.82	0.82	0.00	0.00	16.21	0.89	0.56	0.03	0.52	0.03
250	15.23	0.84	14.30	0.79	14.84	0.82	7.60	0.42	15.96	0.88	0.40	0.02	0.68	0.04
300	16.34	0.90	16.06	0.88	14.70	0.81	8.52	0.47	17.01	0.94	0.76	0.04	0.19	0.01
350	15.04	0.83	17.67	0.97	13.98	0.77	0.00	0.00	17.76	0.98	13.82	0.76	0.35	0.02
400	19.24	1.06	12.82	0.71	12.71	0.70	6.91	0.38	19.25	1.06	0.00	0.00	0.00	0.00
450	18.94	1.04	16.15	0.89	15.49	0.85	10.69	0.59	19.03	1.05	0.06	0.00	0.03	0.00
500	13.30	0.73	14.22	0.78	13.66	0.75	6.91	0.38	14.90	0.82	2.50	0.14	1.43	0.08
550	18.86	1.04	17.67	0.97	13.25	0.73	6.91	0.38	19.13	1.05	0.31	0.02	0.00	0.00
600	19.94	1.10	16.23	0.89	14.45	0.79	10.60	0.58	19.97	1.10	0.02	0.00	0.00	0.00

Oligotrophication (OR) and mineralization (MR) rates (Table 2) along the incremental distance from the glacier forefront manifested almost constant values (except for some outliers at 350 m for OR and 50 m for MR) and no significant regression trend.

### 3.4 Fungal metagenome and succession

The metabarcoding libraries yielded 3,636,481 paired reads. After the removal of the low‐quality sequences, chimaeras, nontarget taxa (plants, Chromista, Protista, Protozoa, and Metazoa), and rare sequence types, we retained 1,305,772 sequences that following normalization were grouped in 855 operational taxonomic units (OTUs, Supporting Information Table S2). Sequencing depth ranged from 12,218 to 128,650. Subsampling was carried out at 10,000 units (~80% of minimum sequencing depth) and the number of obtained OTUs per sample ranged from 11 to 249.

Individual sample rarefaction curves showed a rather small diversity loss at the subsampling threshold, suggesting that sequencing effort and subsampling cutoff were both appropriate (Supporting Information Figure S2). The species accumulation curve showed that sampling effort was sufficient in order to provide an accurate description of the diversity at the sampling location (Supporting Information Figure S3).

**Figure 8:**
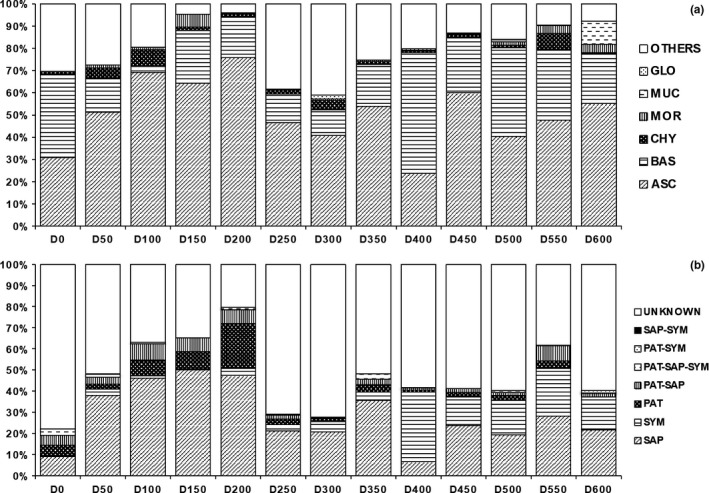
Fungal community composition in terms of relative abundance at the phylum level (a) and at the trophic mode level (b). The sample name (e.g., D100) identifies the distance from the glacier forefront at which the sample was collected (e.g., D100 = 100 m). Abbreviations: ASC: Ascomycota; BAS: Basidiomycota; CHY: Chytridiomycota; GLO: Glomeromycota; MOR: Mortierellomycota; MUC: Mucoromycota; PAT: Pathotrophs; SAP: Saprotrophs; SYM: Symbiotrophs

Sequences were mainly affiliated to Ascomycota (50.7% of relative abundance), Basidiomycota (24.2%), Chytridiomycota (2.6%), Mortierellomycota (1.5%), and Mucoromycota (0.9%). Less than 1% sequences were classified as Glomeromycota, Monoblepharidomycota, Olpidiomycota, and Rozellomycota. The remaining 19.9% were unclassified Fungi (see Figure 8 for differences in composition at the phylum level between samples collected at an incremental distance from the glacier forefront).

**Figure 9:**
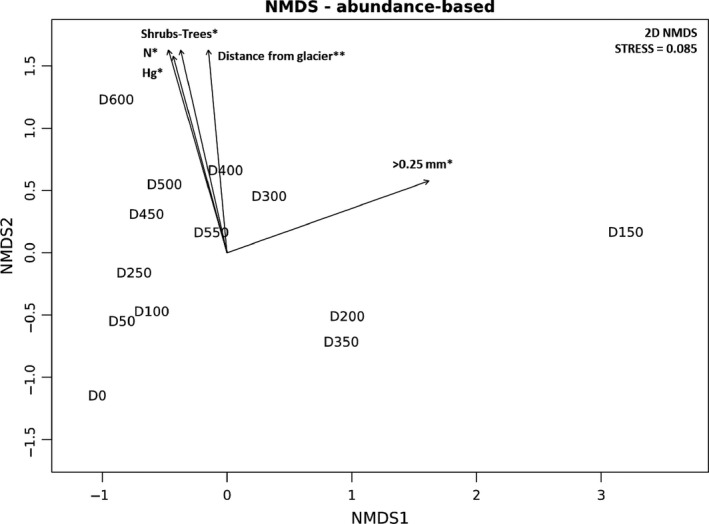
Nonmetric Multidimensional Scaling ordination of using the OTU‐based Bray–Curtis dissimilarity matrix calculated on square‐root/Wisconsin standardized fungal read counts. The fitting of significantly (*0.010 < *p*‐value < 0.050, **0.001 < *p*‐value < 0.010) correlated environmental variables is plotted. The sample name (e.g., D100) identifies the distance from the glacier forefront at which the sample was collected (e.g., D100 = 100 m). Abbreviations: >0.25 mm: soil granulometry fraction higher than 0.25 mm; Hg: mercury; N: total nitrogen

**Figure 10:**
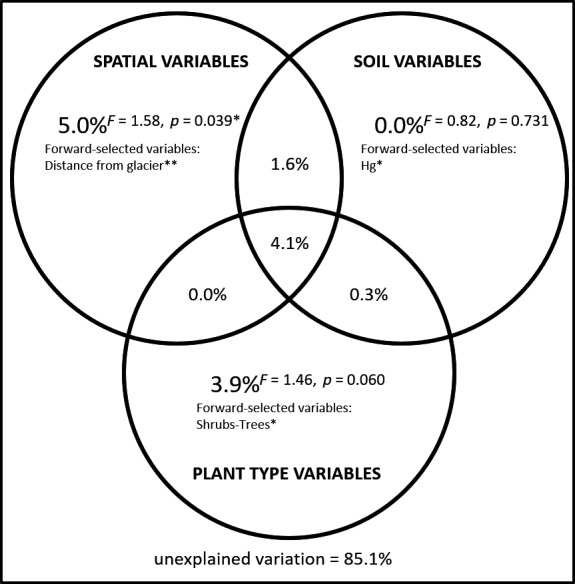
Fungal community variance partitioning between the forward‐selected sets of spatial variables, soil variables, and plant type variables. The fractions of variance explained by the forward‐selected measured environmental variables and the residual variance are reported as adjusted *R* ^2^ (transformed into percentages). The level of significance of the explained fractions according to the modified *F* test for multivariate datasets implemented in the function *forward.sel* of the package *packfor* in R v3.2.0 is also reported. The environmental variables that were forward‐selected using adjusted *R* ^2^ and *α* = 0.05 as cutoffs are listed and their individual statistical significance levels reported (*0.010 < *p*‐value < 0.050, **0.001 < *p*‐value < 0.010)

The NMDS ordination shows that fungal communities distributed along an environmental gradient where the distance from the glacier forefront, the occurrence of shrubs and trees, and the soil Hg and N content are important determinants (Figure 9). This was further confirmed by the variance partitioning analysis which reports that ~15% of fungal community variance could be jointly explained by three variables, that is, the distance from the glacier forefront, the Hg soil content, and the occurrence of shrubs and trees (Figure 10). In particular, two fractions of the variance of 5.0 and 3.9% were exclusively explained by the distance from the glacier forefront and the occurrence of shrubs and trees, respectively. The Hg soil content variable was instead found to explain 6.0% of fungal community variance jointly with the other variables (4.6% with both, 1.6% with the distance from the glacier forefront, and 0.3% with the occurrence of shrubs and trees).

**Figure 11:**
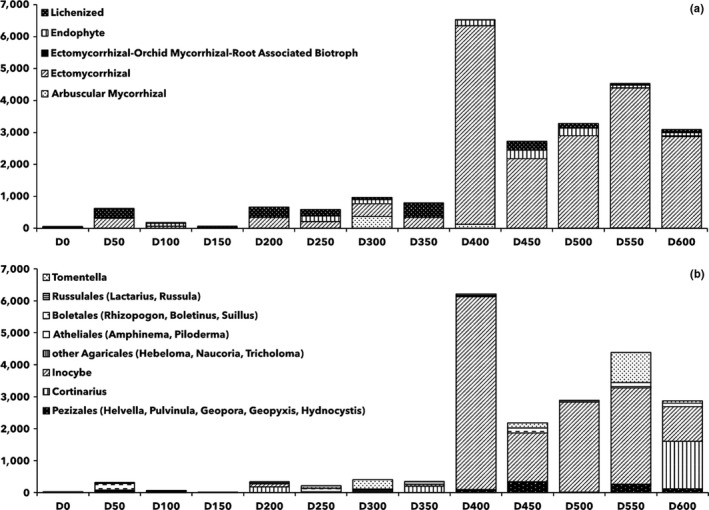
Symbiotroph community composition at the ecological guild level (a) and ectomycorrhizal community composition at the genus level (b). The sample name (e.g., D100) identifies the distance from the glacier forefront at which the sample was collected (e.g., D100 = 100 m)

**Figure 12:**
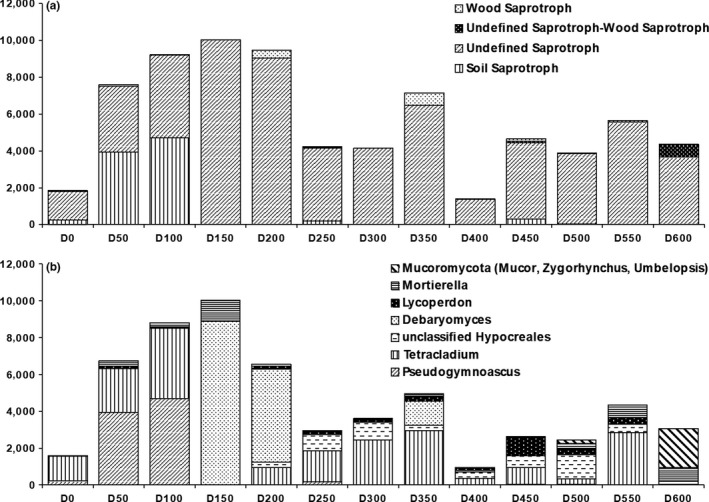
Saprotroph community composition at the ecological guild level (a) and soil and undefined saprotroph community composition at the genus level (b). The sample name (e.g., D100) identifies the distance from the glacier forefront at which the sample was collected (e.g., D100 = 100 m)

Around 45% OTUs could be assigned to a trophic mode and to an ecological guild (Figure 8). Two major trends were characterized at incremental distances from the glacier forefront. First, symbiotrophs (Figure 8) and specifically ectomycorrhizal fungi (Figure 11) were found to increase their relative abundance at more than 350 m from the glacier forefront. The principal fungal genus responsible for this trend was *Inocybe*. Second, higher relative abundances of soil and undefined saprotrophs (Figure 12) were found at shorter distances from the glacier forefront. Here, a strong taxonomic turnover was highlighted, especially among *Tetracladium*,* Debaryomyces*, and *Pseudogymnoascus*.

**Figure 13:**
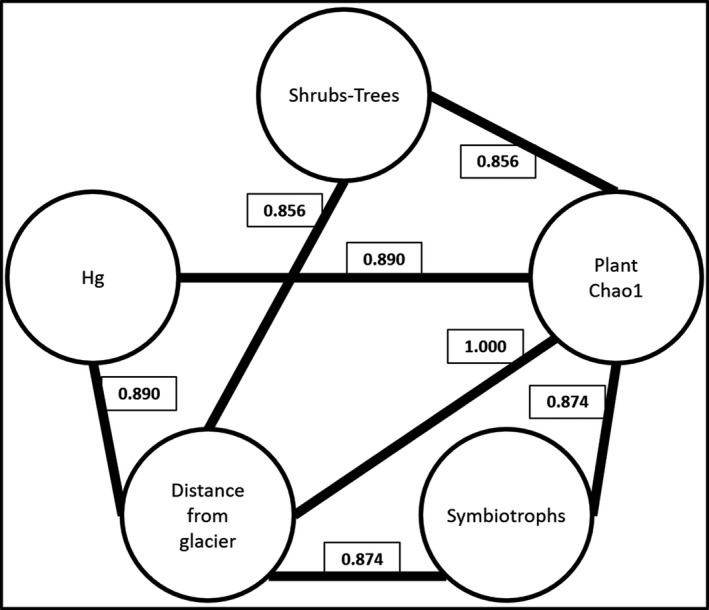
Correlation network computed with fungal trophic modes, plant types and diversity indices, soil variables, and the distance from the glacier forefront as input. Spearman's correlation coefficients were calculated for all combinations of variables and false‐discovery rate correction was applied on the Spearman's correlation *p*‐values. Only correlations that were significant at *α* = 0.01 are shown. Spearman's correlation coefficients are reported within the boxes connecting the correlated variables

The correlation study computed with fungal trophic modes, plant types and diversity indices, soil variables, and the distance from the glacier forefront as input resulted in a network of seven highly significant positive correlations among soil Hg content, the distance from the glacier forefront, the plant diversity index Chao 1, the occurrence of symbiotrophs, and the occurrence of shrubs and trees (Figure 13).

## 4 DISCUSSION

In this paper, we analyzed the last 50 years of deglaciation of the Aktru glacier, which is evident from the analysis of satellite images and the presence of benchmarks left by researchers and explorers time‐by‐time. In particular, we focused on the primary ecological succession revealed by the ice melting of the first 600 m from the glacier forefront, which left empty soil for the development of new ecological communities. According to our measurements, the glacier lost about 12 m per year during the last 50 years.

Plant succession shows clear signs of changes along the incremental distance from the glacier forefront. The first 120 m, corresponding to about the last 10 years of deglaciation, is being populated by 33 primary plant species, although the first 20 m are completely devoid of plants (but populated by some microorganism species, see below). Moreover, only 10 species account for most of the abundance in this part of the primary succession and *Saxifraga oppositifolia* takes over 60% of total herbs abundance. Moving forward along the succession we documented an increase up to 300 m and then a decrease in herbs abundance, in a point where tree species begin to be more frequent. Since 450 m from the glacier forefront, we encountered higher trees (32.7 ± 5.6 cm) of the genera *Salix*,* Myrcaria,* and *Juniperus*. Moss species number is almost homogeneous along the succession, even there is an increase in moss abundance along with the increase in tree species toward the end of our transect (500–550 m). The high positive correlation between the total species richness and the distance from the glacier forefront confirms the evident trend of an ongoing primary succession that tends to an increase in diversity toward late seral stages. Total abundance, instead, shows an hump‐shaped curve more than a linear increasing trend and this confirms that the total abundance is high in the intermediate seral stages of a primary succession but declines in the late stages, with increasing species richness (Odum, 1983).

The analysis of the α‐diversity confirmed an expected increase in plant diversity along the primary ecological succession with increasing distance from the glacier forefront. The values reached at the sampling limit of 600 m by both Shannon and Simpson indices are indicators of a community that composed by an intermediate level of diversity (Cazzolla Gatti, Vaglio Laurin, & Valentini, 2017; Magurran, 2007; Messina et al., 2016). In fact, the approximation derived from the Chao 1 index, as a proxy of the true diversity the studied community could reach the climax stage of the succession, shows that 144.47 ± 25.93 (i.e., 118–170) species could represent the “effective” number of species. This means that comparing this value with our total number of collected species (*n *= 86), from 32.54 to 84.40 other species could be potentially added to the succession.

Moreover, the matrix of shared species (β‐diversity) reveals two main blocks along incremental distance from the glacier forefront (with a threshold of a minimum shared number >2), one between 30 and 150 m (we do not find any species from 0 to 20 m, thus shared number is 0) and another from 110 to 600 m. These two main groups of shared species overlap between 110 and 150 m. This could mean that besides the internal similarity of both the initial and the final successional stages, at an intermediate distance from the glacier forefront, another intermediate successional stage could correspond. The analysis of complementarity (carried out by Morisita–Horn β‐diversity measure and a threshold of complementarity >0.4) confirmed the hypothesis of the presence of three main stages of the plant succession: (a) initial (pioneer species) from 30 to 100 m; (b) intermediate (*r*‐selected species) from 110 to 120–150 m; and (c) final (K‐selected species) from 150 to 550. A similar successional differentiation was observed in the Alps by Tscherko, Hammesfahr, Zeltner, Kandeler, and Böcker (2005). Only species at 550 and 600 m show a low level of complementarity (0.28) among all pairs of successive sampling points. This might confirm that vegetation at 600 m is significantly different from the previous successional stages and could represent the beginning of a new seral community, more represented by trees and less by herbs (toward a potential climax stage).

This study also shows that saprotrophic communities of fungi are widely distributed in the glacier retreating area with higher relative abundances of saprotroph ascomycetes at early successional stages. Here, a strong taxonomic turnover was highlighted, especially involving *Tetracladium*,* Debaryomyces*, and *Pseudogymnoascus*. Saprotrophs play a key role in the cycling of nutrients and litter decomposition. During this process, these fungal components are essential to establish an organic pool in soil with rapid turnover (Semenova et al., 2016). It is suggested that building a complete system of decomposed biological residues for a mature ecosystem may be a necessary task during early primary succession.

Our study limit of 600 m reached the beginning of the climax stage of the primary succession (a riparian forest that has grown in the downhill valley). The presence of relevant water flows and geological movements (high winter precipitations as snow and rainfall, streams formed by summer deglaciation, formation of moraines, landslides, etc.) could represent significant and influencing drivers of the local biodiversity patterns (Cazzolla Gatti, 2016a; Cazzolla Gatti, 2017), as evidenced by other studies (Jones & Henry, 2003). Nevertheless, the ecological plant succession is clearly evident along the deglaciation chronosequence.

Even though the study‐site topography could change quickly, as also confirmed by the absence of a significant patter in the soil granulometry, the evolution of a primary succession is not only evident for plants but also for soil elements and CO_2_ emission and respiration. In fact, both soil nitrogen and carbon show a slight increase toward late seral stages of the succession, with lowest (although non‐negligible) concentrations at the glacier forefront and highest at the successional limit of our study (600 m). The increase in soil C and N toward late seral stages is surely due to the documented higher presence of plants, microfungi, and bacteria, which are able to store these nutrients in the soil, activating the related cycles. The opposite decreasing trend of soil calcium, which shows a reduction in concentration moving away from the glacier forefront, suggests that in the late seral stages of the succession the effect of rock‐weathering that releases calcium in the soil is reduced and compensated by the absorption of organic Ca by plant, fungi, and microorganisms.

We also analyzed mercury's soil concentration as a proxy of soil exposure to the air and atmospheric agents, since mercury reaches the deglaciated soil primarily through atmospheric deposition, both wet and dry and, carried out by water, and accumulates into the free‐of‐ice soil (Krabbenhoft & Schuster, 2002). There are not many important sources of atmospheric mercury in the close proximity of the Aktru glacier, except for some industrial areas in the cities nearby (Warren, 1978), but we show that the exposure time (as increasing distance from the glacier forefront) of the deglaciated soil is related to mercury concentration. The positive highly significant correlation between Hg concentration and distance, besides confirming the successional trend and last 50 years of deglaciation, indicates that even without important emitters, this element might accumulate in the soil in only 50 years up to reach 18–20 ng/g, as we found at a distance of 600 m from the glacier forefront.

The positive increment of soil CO_2_ emission and respiration moving toward later successional stages that we documented at the Small Aktru glacier confirms the well‐known theory of ecosystem succession by Odum (1969). This theory suggests that respiration increases with age and that the difference between carbon uptake and release declines with age primarily because respiratory losses increase.

A successional trend is also clear from microbiological analyses, where the number of Colony‐Forming Unit (CFU) and, in particular, eutrophic and culturable bacteria together with microfungi increase significantly along with the distance from the glacier forefront. In the first 50 m, the number of bacteria is low but microorganisms are not completely absent, even with low soil nutrient levels. Between 50 and 250 m, the number of CFU slightly increases, but at a distance of 300–600 m the growth is 100%–500% higher than at the initial stages. The combination with the higher growth of plant in the intermediate seral communities and the availability of higher quantity of nutrients, seems to catalyze (Cazzolla Gatti, Hordijk, & Kauffman, 2017) the increase in both plant and microorganism abundance and provides support to the facilitation models of primary succession observed by Tscherko et al. (2005) for the microbial community of a deglaciated alpine terrain.

A positive trend in the live/dead assay confirms that there is an increase in the analyzed microorganism abundance along the succession toward late seral stages. However, microorganism diversity remains almost stable along the whole succession, as proved by the absence of a significant trend of the AWCD. This, as also reported by other previous studies (Nemergut et al., 2007; Schmidt et al., 2008), could mean that along the succession there is a microorganism replacement that does not change significantly their species richness, even though their abundance is positively correlated with the successional time. In contrast, fungal diversity in terms of both OTUs and genera fluctuates significantly with a trend where there are several intermediate stages along the succession, in which diversity is high, that are interspersed by samples that have high dominance of few taxa with a lower diversity.

Several studies have previously demonstrated that fungal community shifts are tightly linked to the establishment of plants during soil development process (Brown & Jumpponen, 2014; Dong et al., 2016; Tian et al., 2017). At the Small Aktru glacier, ectomycorrhizal fungi were found to partially replace saprotrophs at a distance from the glacier forefront higher than 350 m. Mycorrhizal fungi are responsible for most nutrient uptake by the majority of land plants and are increasingly recognized as important drivers of terrestrial ecosystem processes (Mohan et al., 2014). As diverse plants species start to establish in the in the earliest colonized sites organic matter slowly accumulates in the soil and microbial and mycorrhizal fungal populations increase and become dominant over time. In line with previous studies (Fujiyoshi et al., 2011; Jumpponen, Trappe, & Cázares, 2002), taxonomic shifts and diversity increased among the ectomycorrhizal taxa, especially at late successional stages (550–600 m distance from the glacier forefront). However, a single genus, that is, *Inocybe*, was frequently dominant despite the turnover in the host–plant community composition and major shifts in soil properties including nutrient status and physical characteristics. This genus was recognized as one of the first colonizers in a primary succession on Mount Fuji in Japan (Nara, Nakaya, & Hogetsu, 2003). More similar to our case, Jumpponen et al. (2002) regularly found sporocarps of the species *Inocybe lacera* under *Salix* spp. at late successional stages in the retreating Lyman glacier (Washington, USA).

Finally, the growth of microfungi is significant and seems fundamental to the development of the succession. Nevertheless, we did not see a relevant change in the number of oligotrophic bacteria and actinobacteria. Their per cent proportion is within the average of those of eutrophic and culturable bacteria (~15%), but their stability during time shows that these groups of microorganisms are a constant presence in any seral community of the ecological succession over the Aktru glacier retreatment.

## 5 CONCLUSION

In this article, we reported and discussed the results obtained from a survey of plants, soil elements, and microorganisms along a chronosequence of deglaciation in the first 600 m of the Maliy Aktru glacier's forefront. Our analysis showed that a clear ecological succession is evident in both plant and microorganism communities, and for soil microelements. The development of an integrated ecosystem during the time since glacier melting is a phenomenon that strongly modifies the landscape and the biogeochemical cycles. Supporting satellite analysis, ground surveys confirm both the entity of deglaciation and the ecological changes undertaken by the bare soil. Besides the basic ecological interest in the theoretical and empirical research on primary successions, whose an almost unique possibility of studying has been recently offered by the disappearance of glaciers worldwide, the development of biological communities and the variation in geochemical parameters represent an irrefutable proof that climate change is altering soils that have been long covered by ice.

Although most of the criticism about the ecological effects of deglaciation arises because of the impossibility to replicate the sampling on something that is unique, as a glacier, the addition of the evidence of a new ecological succession at the Maliy Aktru, Altai Mountains (Russia), provides unchallengeable support to previous studies on other glaciers of Earth. In this regard, the scientific community is producing a great effort in solving the pseudoreplication problem (invoked by Hurlbert, 1984) by showing that replicates (most of the glaciers in the world) under the same treatment (climate change) exhibit similar trends. A fact that cannot be ignored by skeptical scientists and policymakers, particularly in this era of severe anthropogenic impacts on the ecosystems (Cazzolla Gatti, 2016b; Vaglio Laurin et al. 2016).

## CONFLICT OF INTEREST

None declared.

## AUTHOR CONTRIBUTIONS

RCG conceived and designed the experiments. RCG, AD, AIV, and IVV performed the experiments, collected, and analyzed the plant data. AL and AD analyzed the soil samples. IVL, AVP, RCG, and AD analyzed the microbiological samples. SV, EL, and AB analyzed the metagenome and performed the DNA extraction, PCR amplification, and amplicon sequencing. RCG wrote the manuscript; all authors provided editorial advice.
