# Supplementary material for: The last 50 years of climate‐induced melting of the Maliy Aktru glacier (Altai Mountains, Russia) revealed in a primary ecological succession
Source: Ecol Evol. 2018 Jul 2;8(15):7401–20. doi: 10.1002/ece3.4258 (PMC6106165; doi:10.1002/ece3.4258)
Supplement: Supplementary file 1 [file ECE3-8-7401-s001.docx]

**Supporting Information**


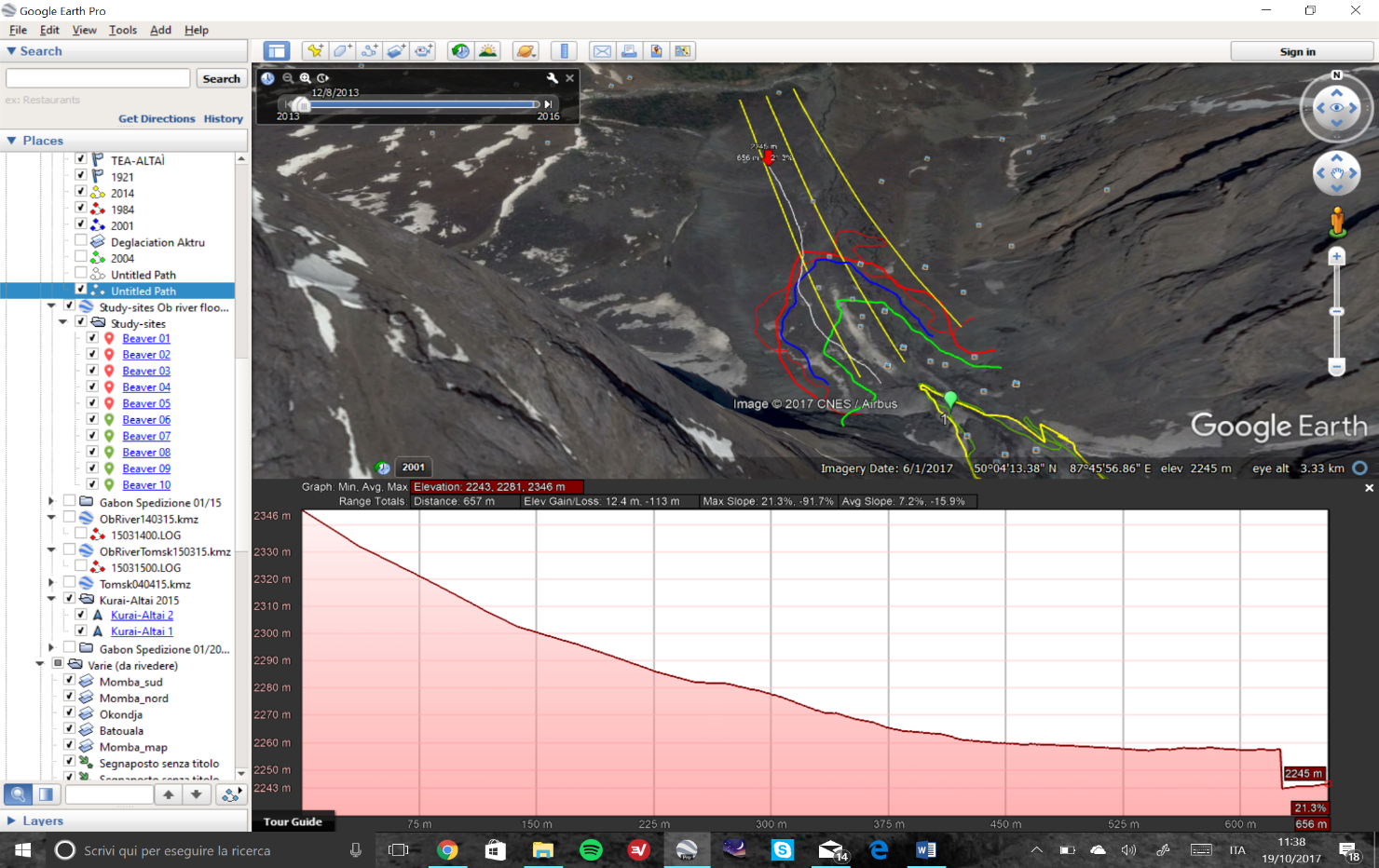


**0**


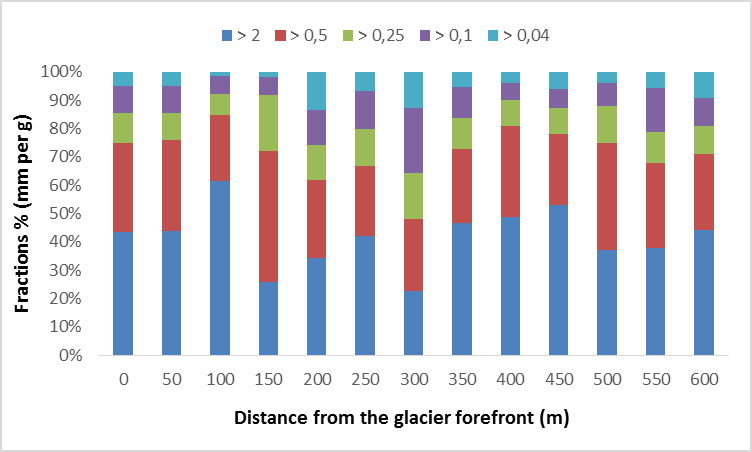


**Supplementary Figure 1** Histograms of soil granulometry (fraction in % as mm per g) along incremental distance from the glacier forefront and, below, the elevational profile.

**
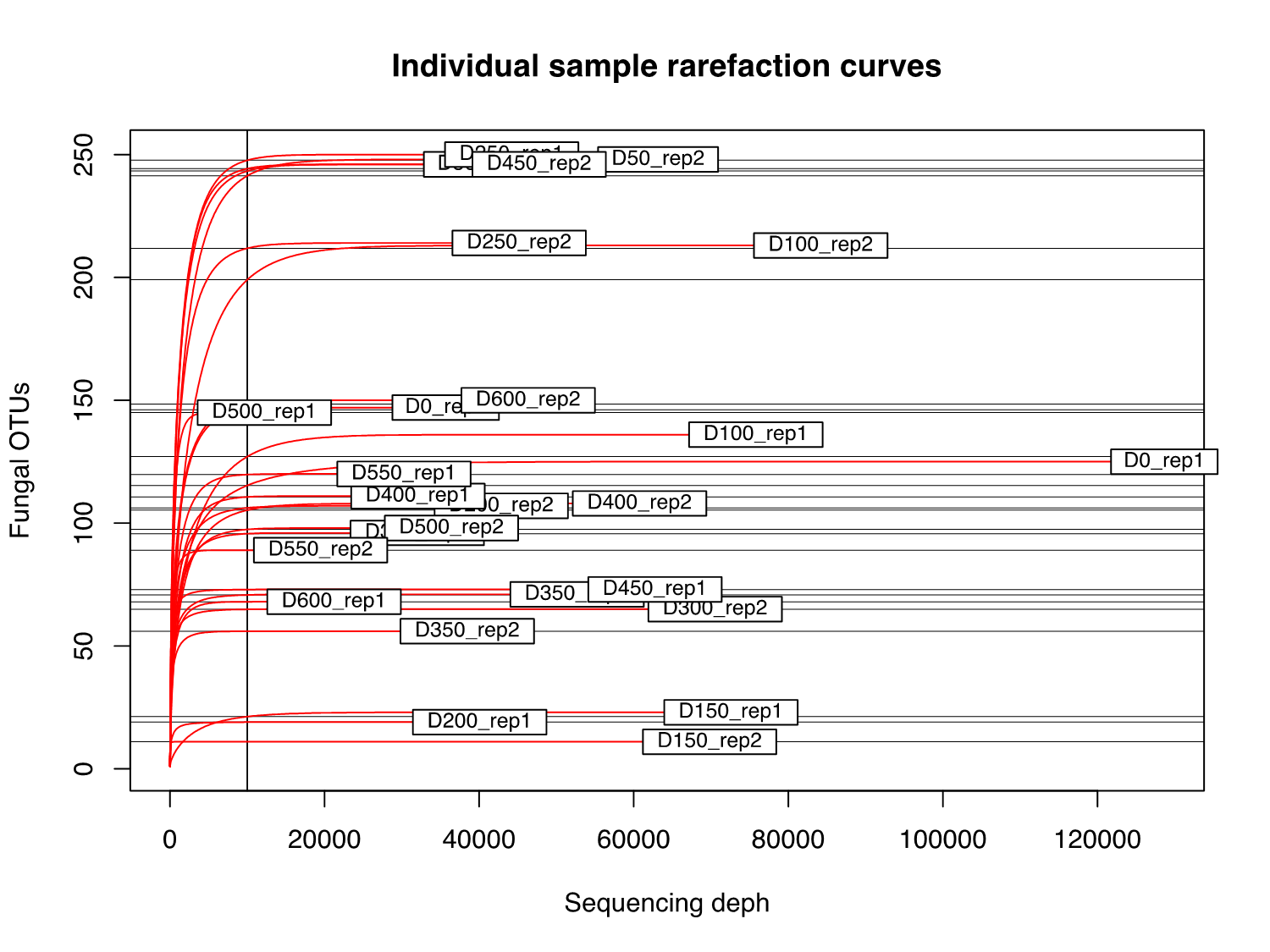
**

**Supplementary Figure 2.** Individual rarefaction curves carried out using the fungal dataset before subsampling. The black vertical line indicate the subsampling threshold that was set at 10,000 units. Horizontal gray lines indicate the number of fungal OTUs that were kept for each sample after subsampling. The sample name (e.g., D100_rep1) identifies the distance from the glacier forefront at which the sample was collected (e.g., D100 = 100 m) and the technical replicate (replicate 1 or replicate 2).


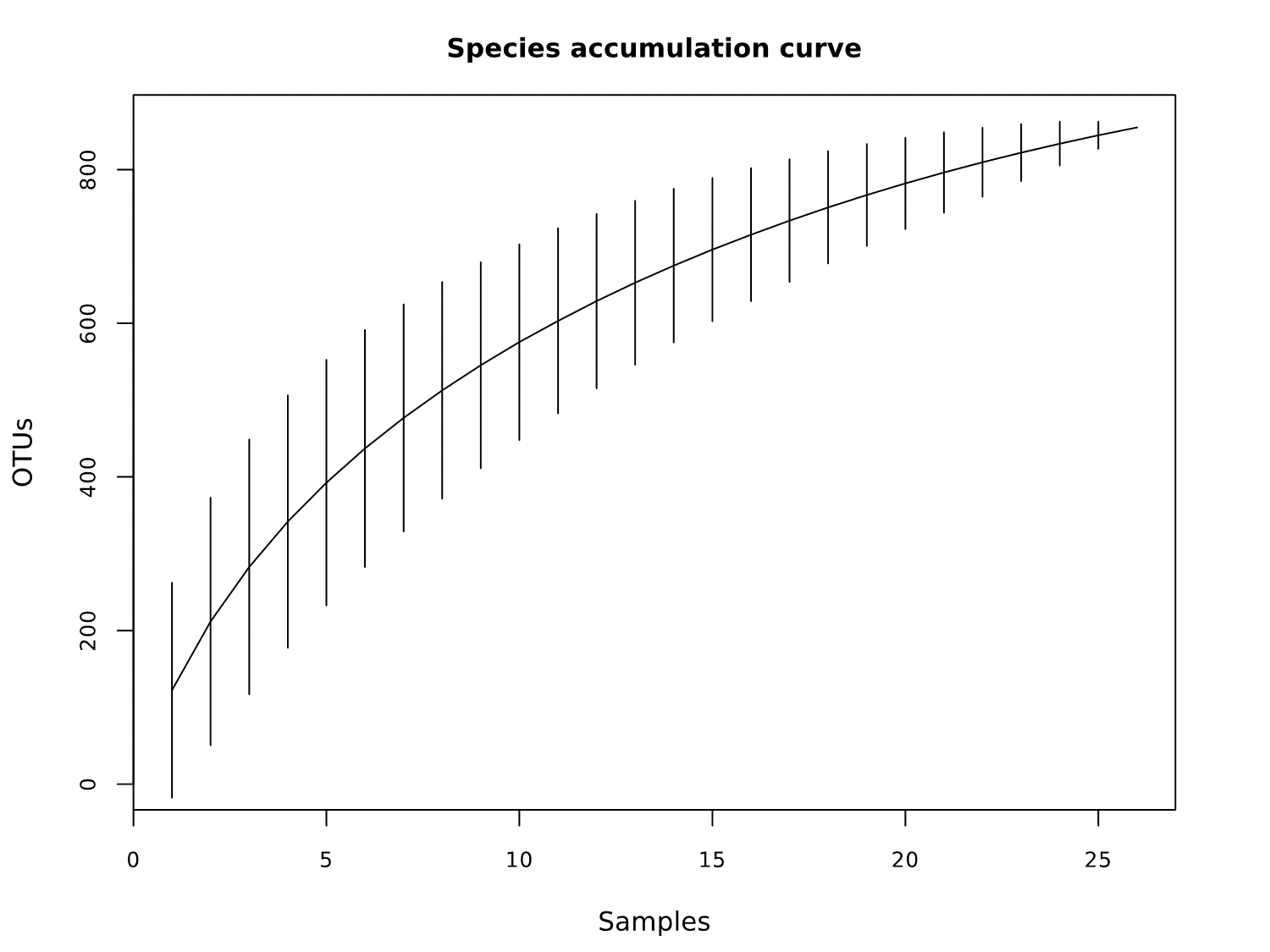


**Supplementary Figure 3.** Species accumulation curves computed with the whole fungal dataset after subsampling at 10,000 units.

**Supplementary Table 1.** List of substrates used for metabolic testing (Ecoplate) in this study

|  | **Carbon Sources** | **Formula** |  | **Carbon Sources** | **Formula** |
| --- | --- | --- | --- | --- | --- |
| N° of cells | **Amino Acids** |  | N° of cells | **Carboxylic Acids** |  |
| 30 | Glycyl-L-Glutamic Acid | C_7_H_12_N_2_O_5_ | 23 | α**-** Keto Butyric Acid | C_4_H_6_O_3_ |
| 25 | L- Arginine | C_6_H_14_N_4_O_2_ | 17 | D- Galactonic Acidy-Lactone | C_6_H_10_O_6_ |
| 26 | L- Asparagine | C_4_H_8_N_2_O_3_ | 18 | D- Galacturonic Acid | C_6_H_10_O_7_ |
| 27 | L- Phenylalanine | C_9_H_11_NO_2_ | 14 | D- Glucosaminic Acid | C_6_H_13_NO_6_ |
| 28 | L- Serine | C_3_H_7_NO_3_ | 24 | D- Malic Acid | C_4_H_6_O_5_ |
| 29 | L- Threonine | C_4_H_9_NO_3_ | 19 | 2- Hydroxy Benzoic Acid | C_7_H_6_O_3_ |
|  |  |  | 20 | 4- Hydroxy Benzoic Acid | C_7_H_6_O_3_ |
|  | **Amines** |  | 21 | γ- Hydroxy Butyric Acid | C_4_H_8_O_3_ |
| 31 | Phenylethylamine | C_8_H_11_N | 22 | Itaconic Acid | C_5_H_6_O_4_ |
| 32 | Putrescine | C_4_H_12_N_2_ | 2 | Pyruvic Acid Methyl Ester | C_4_H_6_O_3_ |
|  |  |  |  |  |  |
|  | **Carbohydrates** |  |  | **Phosphate-Carbon** |  |
| 8 | α- D- Lactose | C_12_H_22_O_11_ | 16 | D,L- α- Glycerol Phosphate | C_3_H_9_O_6_P |
| 9 | β- Methyl-D- Glucoside | C_7_H_14_O_6_ | 15 | Glucose-1- Phosphate | C_6_H_13_O_9_P |
| 7 | D- Cellobiose | C_12_H_22_O_11_ |  |  |  |
| 12 | D- Mannitol | C_6_H_14_O_6_ |  | **Polymeric Compounds** |  |
| 10 | D- Xylose | C_5_H_10_O_5_ | 5 | α**-** Cyclodextrin | C_36_H_60_O_30_ |
| 11 | i- Erythritol | C_4_H_10_O_4_ | 6 | Glycogen | (C_6_H_10_O_5_)_n_ |
| 13 | N- Acetyl-D- Glucosamine | C_8_H_15_NO_6_ | 3 | Tween 40 |  |
|  |  |  | 4 | Tween 80 |  |
